# *Acta E* transforms from *Structure Reports Online *to *Crystallographic Communications*

**DOI:** 10.1107/S1600536814011775

**Published:** 2014-06-10

**Authors:** S. Samar Hasnain

**Affiliations:** a Max Perutz Professor of Molecular Biophysics and Editor-in-chief of IUCr Journals, University of Liverpool, Crown Street, Liverpool, Merseyside L69 7ZB, United Kingdom

**Keywords:** crystallography, editorial, Acta E

At the start of the millennium, the IUCr decided to launch its first fully electronic journal, *Structure Reports Online* (*Section E* of *Acta Crystallographica*), with its founding Main Editors William Clegg (Newcastle, UK) and David Watson (Cambridge, UK). The first issue appeared on 1 January 2001 reporting crystal structure results across a wide range of inorganic, organic and metal-organic materials in a richly hyperlinked HTML format with access to the complete diffraction data, the submitted CIF, and the full checking output. As the only IUCr journal publishing short format articles for small molecule structures, *Acta E*, with its high standards of validation, proved very popular in the community. In 2008, the journal became open access. In order to make open access affordable to authors, the format of published papers was shortened, resulting in large numbers of papers being published, averaging around 4000 per annum in the five-year period 2008–2012. During this period, discussion of structure, its scientific significance or value to the wider community was barely presented in the published paper. This went against the vision of the founders of *Acta Cryst.* Paul Ewald, the founding Editor wrote in his inaugural editorial in 1948 ‘the results of crystal investigations should be presented to physicists, chemists, mineralogists, metallurgists and biologists in a form which enables them to grasp readily the implications for their sciences’. Ewald went on to write 66 years ago, ‘it is essential that the methods by which the results have been gained, and the data on which they are founded, should be fully published so that they may be subjected to the expert criticism necessary to assess their reliability’. This remains true even today. All of the *Acta Cryst.* journals must strive to keep this balance of high-quality data and structures with clear commentary on the scientific significance of the structural results to at least the relevant scientific community.

The year 2014 has seen major changes across all IUCr journals, designed to ensure that the widest possible range of science that relies on structural information is reported. Both *Acta B* and *Acta C* have undergone a major change with significant strengthening of the editorial boards. As many of our readers will know, *Acta B* is now called *Structural Science, Crystal Engineering and Materials* while *Acta C* is now simply called *Structural Chemistry*. Both of these journals have already published a number of special issues, which have begun to define their broadened scope. These journals will continue to expand into new areas, covering all sub-disciplines that come naturally under their titles. We thus expect their impact and influence to grow over the coming decade. There will certainly be some overlap, as is the case for many of the other major families of journals, whether they belong to a commercial publisher (*Nature* and its family) or academic societies such as the American Chemical Society. It is important for us not to have gaps while providing choice to authors with some overlaps among our journals. 

*Acta Cryst. E*, to be called *Crystallographic Communications* from January 2015, will be served by the five Main Editors. Luc Van Meervelt (Leuven, Belgium) joins Bill Harrison (Aberdeen, UK), Helen Stoeckli-Evans (Neuchâtel, Switzerland), Edward Tiekink (Kuala Lumpur, Malaysia) and Matthias Weil (Vienna, Austria) to complete the board of Main Editors. A prestigious International Editorial Advisory Board consisting of Stuart Batten (Melbourne, Australia), Gautam Desiraju (Bangalore, India), Larry Falvello (Zaragoza, Spain), Santiago Garcia Granda (Oviedo, Spain), Judith Howard (Durham, UK), Simon Parsons (Edinburgh, UK), Ian Williams (Hong Kong, China) and Chen Xiao-Ming (Guangzhou, China) will guide the journal. The commitment of the IUCr and the community to this journal is clearly reflected by those who have agreed to serve as Main Editors and on the Editorial Advisory Board. In addition, the journal will continue to be served by an international board of experienced Co-editors who work extremely hard to ensure that the journal publishes quality papers while making the experience of publishing as easy and rewarding as possible for authors.

*Crystallographic Communications* will publish two article types: a longer format than at present (Research Communications) and a short format (Data Reports) that is similar to current articles. Both of these will report crystal structure results across a wide range of materials with a clear obligation on authors to highlight the science behind the structures. We expect Research Communications and Data Reports to be covered by the Science Citation Index and the Data Citation Index, respectively. The comprehensive actions taken as part of the relaunch give us confidence that by the end of 2015, indexation will occur and the journal will be well on the way to having a respectable impact factor within 3 years. 

Each submission will be pre-screened by a Main Editor before being allowed to proceed for peer review. The Main Editors, working with the Managing Editor, will assign a Co-editor in the appropriate category after the successful pre-screening of a manuscript. Measures have been introduced to reduce self-citation and restrict the total number of papers that any author can publish in a year.

A key step in realising our vision for the new *Acta E* has been the development of the Research Communication article format with new text sections designed to help authors bring out the science behind the structures and their determinations. The sections include *Chemical context*, *Structural commentary*, *Supramolecular features*, *Database survey*, *Synthesis and crystallization* and *Refinement*. Authors and readers will be glad to see figures included in the published Research Communication rather than being confined to the Supporting information. For the first time in the journal, individual reports are not limited to single structure determinations. In fact, authors are encouraged to describe the structures of two or more related compounds in a Research Communication. These changes will make *Acta E* the natural home for structure determinations with interesting science to report.

You can see the first articles in the new formats in this issue of the journal, along with new *Notes for Authors*. We look forward to receiving submissions in the new formats from authors old and new from 1 July 2014. *Crystallographic Communications* will remain fully open access so that articles are accessible to all readers around the world irrespective of whether their institutions have access to the journal. The open-access fee remains very modest with waivers for authors where needed.

I together with the distinguished Editorial Advisory Board and the board of Main Editors of the new journal *Crystallographic Communications* look forward to working with existing and new authors to harness the full potential of the structure-based science covered by the new journal through rigour, the quality of the structures and their significance for the broad science communities where these structures make an impact.

